# The renal range of the κ/λ sFLC ratio: best strategy to evaluate multiple myeloma in patients with chronic kidney disease

**DOI:** 10.1186/s12882-020-01771-3

**Published:** 2020-03-31

**Authors:** Alícia Molina-Andújar, Pau Robles, Maria T. Cibeira, Enrique Montagud-Marrahi, Elena Guillen, Marc Xipell, Miquel Blasco, Esteban Poch, Laura Rosiñol, Joan Bladé, Luis F. Quintana

**Affiliations:** 1 Nephrology and Renal Transplantation Department, Hospital Clínic de Barcelona, Universitat de Barcelona, Barcelona, Spain; 2 Institut d’Investigacions Biomediques August Pi I Sunyer (IDIBAPS), Barcelona, Spain; 3 Haematology Department, Hospital Clínic de Barcelona, Institut d’Investigacions Biomediques August Pi I Sunyer (IDIBAPS), Barcelona, Spain; 4 Amyloidosis and Myeloma Unit.Hospital Clínic de Barcelona, Barcelona, Spain; 5 Centro de Referencia en Enfermedad Glomerular Compleja del Sistema Nacional de Salud (CSUR), Barcelona, Spain

**Keywords:** Serum free-light chains, Multiple myeloma, Chronic kidney disease

## Abstract

**Background:**

Monoclonal serum free light chains (sFLC) are a well-known cause of renal impairment (RI) in patients with multiple myeloma (MM). As an indicator of monoclonality, sFLC ratio has acquired a key role in the diagnosis and monitorization of the disease. However, its interpretation is altered in patients with chronic kidney disease (CKD). This study aims to evaluate the modification of the sFLC ratio reference range in patients with CKD, and propose an optimal range for patients with CKD.

**Methods:**

Serum FLC κ/λ ratio and estimated glomerular filtration rate (eGFR) were retrospectively analyzed in 113 control patients (without hematologic disease), 63 patients with MM in complete remission and 347 patients with active MM. The three groups included patients with CKD (eGFR < 90).

**Results:**

In the group of patients without active MM (*n* = 176), the sFLC ratio increased at different stages of CKD without pathological significance, with an increase in the number of false positives specially when eGFR is ≤55 ml/min. An optimal range was established for patients with eGFR ≤55 ml/min/1.73 m2: 0.82–3,6 with maximum sensitivity + specificity for that group with an improvement in the Area under the curve (AUC), 0.91 (0.84–0.97) compared with the current ranges proposed by Katzmann and Hutchinson.

**Conclusions:**

This study confirms the influence of eGFR on the interpretation of the sFLC ratio, showing a decreasing specificity in progressive CKD stages when using the reference sFLC range (Katzmann), especially in patients with eFGR ≤55. According to our results, we suggest a modified optimal range (0.82–3,6) for eGFR ≤55 ml/min/1.73 m2. It is necessary to validate this modified range in larger and prospective studies.

## Background

Monoclonal gammopathies result from a clonal proliferation of plasma cells, secreting monoclonal immunoglobulins as well as immunoglobulin free light chains (FLC), kappa (κ) and lambda (λ), as by-products of immunoglobulin synthesis [1]. This group of diseases include a wide range of clinical entities including light chain systemic amyloidosis, light-chain deposition disease, Waldenström macroglobulionemia and, predominantly, multiple myeloma (MM) [2]. In physiologic conditions, FLC are released into the circulation in small quantities and are rapidly removed by the reticulo-endothelial system but mainly by renal clearance [3]. In MM patients, however, clonal proliferation of plasma cells can result in aberrant production of serum FLC (sFLC), surpassing kidney’s clearance capacity and leading to renal deposition and injury [3]. In MM, renal disease is the most characteristic and extensively studied organ involvement, being cast nephropathy the most frequent injury pattern as well as the predominant cause of renal impairment (RI), i.e. chronic kidney disease (glomerular filtration rate < 60 ml/min/1.73 m^2^), secondary to acute kidney injury (AKI) [4]. In addition, other factors such as age at diagnosis (around the sixth decade), hypercalcemia, dehydration and the use of nephrotoxic drugs, can contribute to the high prevalence of long-standing renal disease in MM, leading to RI in up to 50% of patients at diagnosis, which is also considered an independent poor prognostic factor [5].

Quantitative measurement of sFLC is an essential procedure for diagnosis and monitoring of monoclonal gammopathies. The development of an effective nephelometric assay to quantify the sFLC concentration has optimized the screening and monitoring algorithms of MM [6], using an abnormal κ/λ ratio as an indicator of clonality. However, there is evidence that renal function influences the interpretation of this ratio.

In 2002, Katzmann et al. established a reference “polyclonal” range of 0.26–1.65 for the κ/λ ratio using 282 reference serum samples from the United States of America (USA) [7]. However, quantification of κ and λ sFLC showed a trend towards increasing values with increasing age, which normalized when using the sFLC ratio and when dividing sFLC values by cystatin C. The study hypothesized that the observed increase in sFLC was secondary to a decrease in renal clearance with advancing age.

In 2008, Hutchison et al. described sFLC concentrations and κ/λ ratio in patients with chronic kidney disease (CKD) [8] without MM, demonstrating an increase in the sFLC ratio through increasing CKD stages without presence of monoclonality. As a result, a modified κ/λ ratio for the whole CKD population was suggested: 1.12 (0.37–3.1). This range was applied to a sample of 41 dialysis-dependent MM patients in a second study that same year, showing an improved specificity compared to the standard ratio (93 vs 99%) with no loss of sensitivity [9]. However, this range has not been properly validated to date.

Considering the significant number of MM patients showing RI and its influence on the interpretation of the κ/λ ratio, it might be useful to establish a reliable reference range that guarantees high specificity and sensitivity in patients with renal failure.Therefore, we retrospectively analyzed the sFLC concentrations and estimated glomerular filtration rate (eGFR) of patients with active MM, patients with MM with complete remission (CR) and controls (without hematologic disease) in order to determine the influence of CKD stage on the sFLC ratio and establish an optimal range adjusted by renal function.

## Materials and methods

### Patients and study design

The κ/λ FLC ratio in serum and eGFR was analyzed retrospectively by Chronic Kidney Disease Epidemiology Collaboration equation (CKD-EPI) in 1469 consecutive patients attended in Hospital Clínic of Barcelona between December 2014 and December 2017. Data were retrospectively obtained regarding sFLC, sFLC ratio, renal function (eGFR and serum creatinine), CKD stage and chain isotypes in the active MM group. Two groups were selected: a control group (total *n* = 176) comprising patients with no haemathological disorders including no presence of monoclonal gammopathy of undetermined significance (MGUS) by immunofixation (*n* = 113) or MM with CR (*n* = 63); and a group with diagnosis of active MM (*n* = 347). Patients diagnosed of MGUS or other hematological diseases were excluded. Only patients with stable renal function in the last 3 months were included.

Diagnostic criteria for MM were applied according to the International Myeloma Working Group (2014), defined as the presence of > 10% clonal plasma cells in bone marrow biopsy with: either signs of end-organ damage (CRAB: hypercalcemia ≥2,75 mmol/L mg/dL or ≥ 0,25 mmol/L above normal range, eGFR < 40 mL/min or serum creatinine ≥2 mg/dL, hemoglobin < 10 g/dL or > 2 mg/dL below normal range, ≥1 bone lesion by imaging test), or > 60% clonal plasma cells in bone marrow, or sFLC ratio ≥ 100 or > 1 proven focal lesion by MRI. Solitary plasmocytoma, smoldering MM and MM with partial response to chemotherapy, according to the International Myeloma Working Group criteria (2016), were also included in the active MM group. MM in complete response to chemotherapy, was defined as disappearance of the M-component on serum and urine in inmunofixation, and 5% or fewer plasma cells o bone marrow aspiration. Afterwards, a descriptive transversal study was designed. First, the sensitivity and specificity of the sFLC ratio normal range reported in the literature (0.26–1.65 and 0.37–3.1) [7, 8] was measured in the cohort; second, Receiver Operating Characteristic (ROC) analysis was carried out dividing the population of each of the 2 cohorts in subgroups of different eGFR, establishing the optimal cut-off for the k/λ sFLC ratio and for the eGFR (with maximum diagnostic sensitivity and specificity).

### Laboratory analysis

sFLC levels were assessed by nephelometry, on a Dade-Behring BN II Analyzer, using particle-enhanced, high-specificity, polyclonal immunoassays (Freelite® assays from The Binding Site Ltd., Birmingham, UK) [10]. The reported sensitivity for the sFLC detection of this assay has been < 1 mg/L^7^. The normal serum reference ranges used have been previously reported and were as follows: κ = 3.3 to 19.4 mg/L; λ = 5.7 to 26.3 mg/L; κ/λ ratio = 0.26 to 1.65 [7].

The eGFR was calculated using the CKD-EPI formula: eGFR =141 x min (S_Cr_/κ, 1)^α^ x max (S_Cr_ /κ, 1)^-1.209^ × 0.993^Age^ × 1.018 [if female] × 1.159 [if black].

### Statistical analysis

*IBM SPSS Statistics for Windows, Version 25.0. (Armonk, NY: IBM Corp.), IBM Corp. Released 2017* was used to assess statistical significance. Categorical variables were summarized as frequencies and percentages. All continuous variables had a non-normal distribution, as assessed by Kolmogorov-Smirnov test, and are therefore presented as medians with interquartile range (IQR). Mann-Whitney U test was used to compare the differences in sFLC and sFLC ratio, as well as eGFR and serum creatinine by active MM diagnosis. ROC curve analysis was used to determine the sFLC ratio range with the highest specificity and sensitivity for patients with RI. The area under the curve (AUC) of sensitivity was plotted against 1-specificity and was reported with a 95% confidence interval (CI).

## Results

### Study populations

A total of 523 patients were selected for our study, 347 with active MM and 176 without active MM as control group. When comparing patients with and without active MM (Table 1), sFLC ratio presented an statistically significant difference (*p* < 0.01). Distribution of eGFR and serum creatinine, however, did not show differences between the control group and the group with active MM (*p* = 0.314 and p 0.854, respectively).

**Table 1:** Patients characteristics according to presence of active MM diagnosis. Median creatinine and estimated glomerular filtration rate are not different in both groups. Median κ, λ sFLC and sFLC ratio present statistically significant differences (*p* < 0.01), with higher Kappa and sFLC ratio in patients with active MM . Surprisingly, lambda is increased in the group without MM in comparison with the active MM group, probably because of the higher incidence of Kappa light chain isotype in the MM group. The sFLC ratio corrects this fact

Variables	All patients (***n*** = 523)	Patients with active MM (***n*** = 347)	Patients without active MM (***n*** = 176)	***P***-value
**Median eGFR (IQR)**	85 (62–91)	84(60–91)	86 (65–91)	0,314
**Creatinine (mg/dl) (IQR)**	0,83 (0,67-1,1)	0,84 (0,66-1,12)	0,82 (0,68-1,07)	0,854
**MM Type**				
IgG (%)		198 (57)		
IgA (%)		88 (25,4)		
IgM (%)		6 (1,7)		
IgD (%)		4 (1,1)		
IgG + IgA (%)		6 (1,7)		
Bence-Jones (%)		44 (12,7)		
Non-secretory (%)		1 (0,3)		
**FLC type**				
Κ sFLC (%)		227 (65,4)		
λ sFLC (%)		114 (32,8)		
Κ + λ sFLC (%)		5 (1,4)		
Non-secretory (%)		1 (0,3)		
**FLC level (mg/L)**				
Median k sFLC (IQR)	21,1 (11,5-65,4)	26,4 (10,9–147)	17,35 (12,27-32,12)	< 0.001
Median λ sFLC (IQR)	13,8 (7,5-28,6)	11,2 (6,2-30,4)	16,4 (11,5-26,47)	0,001
Median sFLC ratio (IQR)	1,34 (0,77-6,24)	2,5 (0,49-17,65)	1,11 (0,89-1,39)	< 0.001

*eGFR* Estimated Glomerular Filtration Rate, *IQR* Interquartile range, *MM* Multiple Myeloma, *sFLC* Serum Free Light-Chain

### Reference sFLC range applied to CKD stages

When comparing the sFLC ratio according to CKD stages in the control group (n 176), a progressive increase was observed with worsening CKD stages (Table 2), being more significative in the subgroup of eGFR ≤55 ml/min. Thereby, if we apply the normal range described by Katzmann to that group, we obtain a 37% of false positives. If we describe in more detail this subgroup of patients with eGFR ≤55 ml/min we can observe that median eGFR and creatinine are not statistically different in the active MM group compared with the non-active MM. Kappa light chain and sFLC ratio is higher in the active MM group (Table 3).

**Table 2:** sFLC ratio distribution according to eGFR in the group of patients without active MM. sFLC ratio range increases when there is a decrease in eGFR. The number of false positives according to the the current normal range (Katzmann) also increases specially in patients with eGFR ≤55 ml/min

	eGFR ≥ 90 (***n*** = 76)	eGFR 56–89 (***n*** = 70)	eGFR ≤ 55 (***n*** = 30)
**sFLC ratio (IQR)**	1,04 (0,85-1,3)	1,11 (0,89-1,39)	1,31 (0,95-2,08)
**False positives (%)**	6/76 (7,9)	6/70 (8,5)	11/30 (36,7)

*eGFR* Estimated Glomerular Filtration Rate, *IQR* Interquartile range, *MM* Multiple Myeloma, *sFLC* Serum Free Light-Chain

**Table 3:** Patients’ characteristics according to eGFR > 55 and ≤ 55 ml/min. Median κ and λ sFLC showed statistically significant differences between the 2 groups. Kappa light chain was the most prevalent chain in the two active MM groups

Patients with active MM (***N*** = 347)			Patients without active MM (***n*** = 176)			***P*** value
**eGFR > 55 (*n*** **= 266)**	Median k sFLC (IQR) mg/L	21,35 (10,45-103,25)	**eGFR > 55 (*n*** **= 146)**	Median k sFLC (IQR) mg/L	15,8 (11,14-24,85)	< 0.001
	Median λ sFLC (IQR) mg/L	10,45 (6–23,9)		Median λ sFLC (IQR) mg/L	15,2 (11,15-21,02)	0.001
	Median sFLC ratio (IQR)	2,2 (0,53-14,88)		Median sFLC ratio (IQR)	1,09 (0,87-1,34)	< 0.001
**EGFR ≤ 55 (*n*** **= 81)**	Median k sFLC (IQR) mg/L	57,7 (15,35–815)	**EGFR ≤ 55 (*n*** **= 30)**	Median k sFLC (IQR) mg/L	55,35 (35,47-94,15)	0,001
	Median λ sFLC (IQR) mg/L	17,7 (7,9–149)		Median λ sFLC (IQR) mg/L	35,95 (22,62-62,02)	< 0,001
	Median sFLC ratio (IQR)	5,34 (0,107-71,76)		Median sFLC ratio (IQR)	1,31 (0,95-2,08)	0,001
	Creatinine mg/dL (IQR)	1,6 (1,24-2,68)		Creatinine mg/Dl (IQR)	2,11 (1,43-2,98)	0,108
	eGFR (IQR)	39 (19,5–49)		eGFR (IQR)	28,8 (19,6-50,5)	0,429

*eGFR* Estimated Glomerular Filtration Rate, *IQR* Interquartile range, *MM* Multiple Myeloma, *sFLC* Serum Free Light-Chain

### Optimal adjusted FLC ratio according to eGFR

The optimal renal reference range for sFLC ratio was assessed by ROC analysis to enhance the lack of specificity observed in patients with CKD stage ≥3 when using the reference ratio to diagnose patients with MM. The two study groups were divided according to the severity of renal dysfunction based on the following eGFR levels: < 90, < 60, < 55, < 50, < 45 ml/min/1.73 m^2^. The resulting ratio with maximal sensitivity and specificity was 0.82–3,6 for an eGFR ≤55 ml/min/1.73 m^2^, with 90% specificity and 91,1% sensitivity. Therefore, the eGFR cut-off was stablished at 55 ml/min/1.73 m^2^ instead of 60 ml/min, as it proved to provide a greater statistical power.

**Fig. 1:**
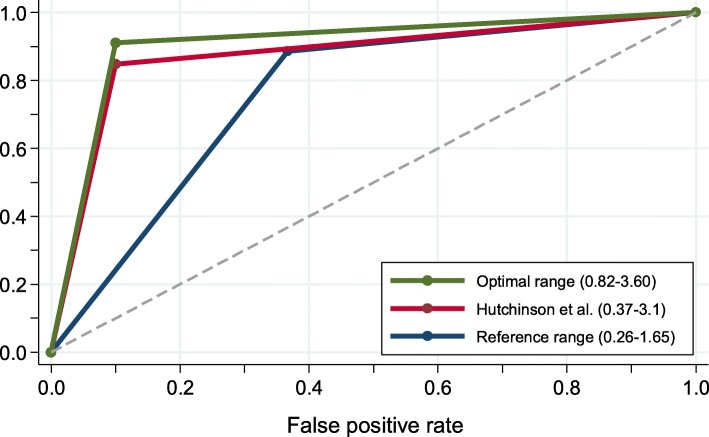
ROC curves diagrams comparing the available sFLC ranges in patients with eGFR ≤55

Comparing the obtained new optimal range with the two ranges described in the literature [the established reference range (0.26–1.65) and the renal range suggested by Hutchison et al. (0.37–3.1)], the new one based on MM patients with an eGFR ≤55 ml/min/1.73m^2^, showed clear superiority by the respective AUC: 0.91 (95% CI: 0.84–0.97) for the new optimal range, 0.87 (95% CI: 0.81–0.94) for the Hutchison et al. range, and 0.76 (95% CI: 0.65–0.95) for the Katzmann [7] reference range (Table 4, Fig. 1); and the respective sensitivities and specificities were 91 and 90%, 84,8 and 90%, and 88,6% and 66,3%. It is important to highlight that our range increases the sensitivity with no loss of specificity in comparison with Katzmann and Huchison.

**Table 4:** κ/λ ratio: AUC, sensitivity, specificity and predictive values; point estimates (95% CIs) for eGFR ≤55

	Optimized (0.82–3.60)	Hutchinson (0.37–3.10)	Katzmann (0.26–1.65)
Sensitivity	91.1 (82.6–96.4)	84.8 (75–91.9)	88,6 (79,5-94,7)
Specificity	90.0 (73.5–97.9)	90 (73.5–97.9)	66,3 (43,9-80,1)
AUC	0.91 (0.84–0.97)	0.87 (0.81–0.94)	0,76 (0.65–0.85)
PPV	96.0 (88.8–99.2)	95.7 (88–99.1)	86,4 (77–93)
NPV	79.4 (62.1–91.3)	69.2 (52.4–83)	67,9 (47,6-84,1)

## Discussion

The purpose of this study was to establish a reliable modified κ/λ ratio in patients with RI as a screening and monitoring test for MM, considering its previously observed influence on the reference ratio described in the literature [8, 9].

The results here obtained confirm a significant increase in sFLC ratio with progressive CKD stages in patients without active MM, leading to a decrease in specificity with declining eGFR when using the reference sFLC range for MM diagnosis, especially in patients with eGFR ≤55 ml/min. In other words, by using the reference κ/λ ratio described in the literature (0.26–1.65), a MM diagnosis would be suspected in a greater number of healthy patients (false positives) as eGFR decreases, since more patients would obtain an above normal ratio**,** leading to a decrease in specificity. These results confirm the need to establish a modified ratio for this population. In fact, the new normal range for the sFLC ratio here obtained 0.82–3,6 for patients with eGFR ≤55 ml/min/1.73 m^2^, substantially improved the AUC when compared to the traditional reference range by Katzmann et al. and by Hutchison et al.

Since the establishment of a reference sFLC ratio by Katzmann et al. [7] in 2002, the influence of CKD in the interpretation of the ratio has remained unclear. In this first study, the increase of sFLC with age was attributed to a decrease in renal function by normalizing it to cystatin C and the sFLC ratio remained within the reference range, thus indicating policlonality variations according to the investigators. That study hypothesized that the observed increase in serum FLC was secondary to a decrease in renal clearance with advancing age. However, the filtration rate of the reference population was not described in the study, and no secondary analysis regarding renal function was conducted.

In a later study [8], an increase in sFLC ratio with progressive CKD stages was observed in a non-MM population, and a modified “polyclonal” range (0.37–3.1) was suggested for patients with CKD. This increase in the κ/λ ratio reflects a change in the dynamics of serum FLC degradation in renal dysfunction. In normal subjects, the clearance of FLC from serum is dominated by renal removal which is more effective for the smaller, monomeric, κ light chain molecules. This is most likely the reason for the shorter serum half-life of κ light chains and therefore a median κ/λ FLC ratio of approximately 0.6 is observed, instead of 1. As the kidney function worsens, however, the reticulo-endothelial system becomes an increasingly important route for FLC degradation [11], which is not influenced by the molecular weight of the FLC, but at much lower rates than those observed through kidney clearance. As a result, serum half-lives for the two FLC increase, approximating to the respective underlying production rate which is almost twice as high for κ FLC as for λ FLC [12]. Therefore, an increase in the sFLC ratio in CKD patients can be explained without the presence of a monoclonal gammopathy.

However, the range suggested by Hutchison et al. (0.37–3.1) has not been properly validated in a substantial and representative sample of patients, and the range was established globally for CKD patients without stratifying them according to the severity of RI. Our findings indicate that, not only the reference ratio should be modified in patients with CKD, but also that an optimal range with increased sensitivity and specificity is reached when establishing a specific cut-off for patients with eGFR ≤55 ml/min.

In clinical practice, the establishment of an optimal sFLC ratio according to the severity of RI has substantial relevance. Monoclonal gammopathies affect as many as 5% of the population by the age of 70 years [13] and the prevalence of patients with CKD stage ≥3 can rise to more than 40% in this population in some European countries [14]. MGUS, characterized by the presence of paraprotein with < 10% bone marrow plasma cells and without end-organ damage, is the most frequent diagnosis among these patients. Interestingly, it is also the precursor form of MM and other paraprotein related diseases with a 1%/year risk of progression, therefore entailing clinical follow-up. Considering the high prevalence of both MGUS and CKD in elderly patients, specifically eGFR ≤55 /ml/min/1.73m^2^, the establishment of an optimal reference range for this subgroup is especially important not only to ensure a reliable suspicion of progression during follow-up of MGUS, but also to decrease the number of false positives thanks to an improved specificity. Without considering the patient’s CKD stage, more patients would fall outside the reference range, therefore, possibly being sent to physician care or undergoing unnecessary tests to assess progression risk, with the resulting medical expenses for hospitals, as well as anxiety produced to patients by communicating a possible diagnosis of a neoplasm. Therefore, we think that by establishing an optimal sFLC ratio range for this population, patients could be stratified more reliably according to the risk of progression and even undergoing their follow-up through outpatient care, without resorting to physician care.

One limitation of the study is that our findings only relate to patients with MM, not with other paraprotein-related diseases. Some patients who do not fulfill criteria of MM can also experience kidney damage due to paraprotein deposition, thus configuring the term monoclonal gammopathy of renal significance (MGRS). This term includes a wide range of monoclonal diseases (light chain amyloidosis, cryoglobulinemic glomerulonephritis, light-chain deposition disease, among others) some of which can coexist with MM and its lesion pattern. Therefore, the assessment for MGRS existence, as well as the application of the proposed sFLC ratio on MGRS patients should be evaluated in future studies. Another possible limitation of this study is the fact that the number of patients in CKD stage V (eGFR ≤15 ml/min/1.73 m^2^) was low (13 in the active MM group and 3 in the control group), resulting in less precision for the results obtained in this subgroup,

## Conclusion

In conclusion, the sFLC k/λ ratio is a reliable method for the screening and monitoring of MM in patients with normal or close to normal renal function but it is necessary to consider the eGFR for its optimal interpretation. In this study, we confirmed that not only the sFLC k/λ ratio increases with progressive CKD stages, but also it becomes more clinically relevant in patients with FGR ≤ 55 ml/min, likely due to the alteration in the degradation rates of the κ and λ FLC and how they are affected by CKD. It has been possible to correct this physiological alteration and establish an optimal sFLC ratio interval of 0.82–3,6 for patients with eGFR ≤55 ml/min/1.73m^2^, resulting in considerably higher specificity + sensitivity than the reference ratio used to date.

It is important to validate this new range in future prospective studies, and its possible utility in patients with MGRS or with monoclonal gammopathies different from MM.

## Data Availability

The datasets used and/or analysed during the current study are available from the corresponding author on reasonable request.

## Abbreviations

AUC: Area Under the Curve
CKD: Chronic Kidney Disease
CKD-EPI: Chronic Kidney Disease Epidemiology Collaboration
CI: Confidence Interval
CR: Complete remission
eGFR: Estimated glomerular filtration rate
FLC: Free light chains
MGRS: Monoclonal gammopathy of renal significance
MGUS: Monoclonal gammopathy of undetermined significance
MM: Multiple Myeloma
ROC: Receiver Operating Characteristic
sFLC: Serum free light chains
USA: United States of America
